# Spatial distribution of iron rich foods consumption and its associated factors among children aged 6–23 months in Ethiopia: spatial and multilevel analysis of 2016 Ethiopian demographic and health survey

**DOI:** 10.1186/s12937-020-00635-8

**Published:** 2020-10-08

**Authors:** Sofonyas Abebaw Tiruneh, Belete Achamyelew Ayele, Getachew Yideg Yitbarek, Desalegn Tesfa Asnakew, Melaku Tadege Engidaw, Alemayehu Digssie Gebremariam

**Affiliations:** 1 Department of Public Health, College of Health Sciences, Debre Tabor University, Debre Tabor, Ethiopia; 2 Wogeda Primary Hospital, Amhara Regional Health Bureau, Bahir Dar, Ethiopia; 3 Department of Biomedical Sciences (Medical Physiology), College of Health Sciences, Debre Tabor University, Debre Tabor, Ethiopia

**Keywords:** Iron rich food, Spatial distribution, Children, Ethiopia

## Abstract

**Background:**

Micronutrient deficiencies are the most prevalent nutritional deficiencies that cause serious developmental problems in the globe. The aim of this study was to assess the spatial distribution of iron rich foods consumption and its associated factors among children aged 6–23 months in Ethiopia.

**Methods:**

The data retrieved from the standard Ethiopian Demographic and Health Survey 2016 dataset with a total sample size of 3055 children aged 6–23 months. Spatial scan statistics done using Kuldorff’s SaTScan version 9.6 software. ArcGIS version 10.7 software used to visualize spatial distribution for poor consumption of iron rich foods. Multilevel mixed-effects logistic regression analysis employed to identify the associated factors for good consumption of iron-rich foods. Level of statistical significance was declared at a two-sided *P*-value < 0.05.

**Results:**

Overall, 21.41% (95% CI: 19.9–22.9) of children aged 6–23 months had good consumption of iron rich foods in Ethiopia. Poor consumption of iron rich foods highly clustered at Southern Afar, Southeastern Amhara and Tigray, and the Northern part of Somali Regional States of Ethiopia. In spatial scan statistics, children aged 6–23 months living in the most likely cluster were 21% more likely vulnerable to poor consumption of iron rich foods than those living outside the window (RR = 1.21, *P*-value < 0.001). Child aged 12–17 months (AOR = 1.90, 95% CI: 1.45–2.49) and 18–23 months (AOR = 2.05, 95% CI: 1.55–2.73), primary (AOR = 1.42, 95% CI:1.06–1.87) and secondary and above (AOR = 2.26, 95% CI: 1.47–3.46) mother’s education level, rich (AOR = 1.49, 95% CI: 1.04–2.13) and middle (AOR = 1.83, 95% CI: 1.31–2.57) household wealth status, Amhara (AOR = 0.24, 95% CI: 0.09–0.60), Afar (AOR = 0.38, 95% CI: 0.17–0.84), and Harari (AOR = 2.11, 95% CI: 1.02–4.39) regional states of Ethiopia were statistically significant factors for good consumption of iron rich foods.

**Conclusion:**

Overall, the consumption of iron rich foods was low and spatially non-random in Ethiopia. Federal Ministry of Health and other stakeholders should give prior attention to the identified hot spot areas to enhance the consumption of iron rich foods among children aged 6–23 months.

## Introduction

Currently, micronutrient deficiencies are the most prevalent nutritional deficiencies that causes serious developmental problems in the world [1]. Anemia is the world most common micronutrient deficiency that affects more than 2 billion people globally. According to the World Health Organization (WHO), in 2011 the Global prevalence of anemia among children was 42.6% [2]. From the global burden, Southeast-Asian (53.8%), Eastern-Mediterranean (48.6%) regions and African (62.3%) account for the high burden of anemia [3]. However, in most parts of the world, children aged 6–23 months are more vulnerable to iron deficiency anemia [4]. Due to higher iron requirement related to growth, infants and young children are primarily affected by anemia [5]. Anemia can adversely affect the health, cognitive development, school achievement, and work performance of individuals [4].

In developing countries, the diets of children aged 6–23 months old are often characterized by low dietary diversity, poor consistency and less nutritious foods. Despite iron requirements are high at age 6–11 months due to rapid growth, infants and children have low-quality diets [6, 7].

According to the 2011 Ethiopian Demographic and Health Survey report, the overall prevalence of anemia among children aged 6–23 months was 60.9% [7]. Some of the adverse health consequences of anemia among pre-school children continue throughout the lifetime [8]. These consequences include impaired cognitive development, poor growth, increased susceptibility to infections, fatigue, and lower physical activity [8].

Despite animal-source foods are rich in protein, fat, and micronutrients, their cost limits the consumption of these foods in Ethiopia. As a result, in a resource-limited setting including Ethiopia do not have the physical access and economic capability to purchase fortified products of animal source foods. Low consumption of animal source foods may result in micronutrient deficiencies such as anemia [6]. According to the recent 2016 Demographic and Health Survey report in Ethiopia, 44% of children aged 6–59 months are anemic and half of them related to iron deficiency. The major causes of iron deficiency are inadequate intake, low bioavailability, and infection [7]. Lack of awareness to iron rich foods, short birth spacing practice, feeding culture, maternal education, household wealth status were some of the factors that affect children’s consumption of iron rich foods [9].

The age younger than two years is a critical window of opportunity for growth and development of the infant and young child. This period is critical and may influence how the children develop, grow, and learn now or later [3, 10]. Therefore, during this time child needs proper nutrition for growth, physical maturity, and emotional and cognitive development.

Despite the above facts, there is no documented evidence of iron rich foods consumption status of children aged 6–23 months in Ethiopia. Therefore, the aim of this study was to assess the spatial distribution of iron rich foods consumption and its associated factors among children aged 6–23 months in Ethiopia.

## Methods and materials

### Data source

The data retrieved from the standard 2016 Ethiopian Demographic and Health Survey (EDHS) dataset. A nationally representative cross-sectional survey was conducted from January 18, 2016, to June 27, 2016, which is available from the standard measure Demographic and Health Survey database website. EDHS 2016 was the fourth nationally representative survey. Ethiopia is situated in the horn of Africa from 3^0^ to 14^0^N and 33^0^ to 48°E.

### Source and study population

All living children aged 6–23 months were the source population, whereas, all selected/sampled living children aged 6–23 months living with their mother were the study population. In 2016 EDHS, a total of 645 clusters (Enumeration Areas) (202 urban and 443 rural) were selected with a probability proportional to each Enumeration Areas size and independent selection in each sampling stratum. Among the total of selected clusters with zero coordinates and clusters not had the proportion of consumption iron rich foods were excluded for the analysis. Finally, a total of 598 (185 urban and 413 rural) clusters were included for this study. In the selected Enumeration Areas (EAs) a total of 3055 weighted number of living children aged 6–23 months living with their mother were included for this study. The recorded data were accessed at www.measuredhs.com.

### Data collection tools and procedures

Ethiopian Demographic and Health Survey data were collected by two-stage stratified sampling. Each region of the country stratified into urban and rural areas, yielding 21 sampling strata. In the first stage, 645 Enumeration Areas selected with a probability proportional to the Enumeration Area size by independent selection in each sampling stratum. In the second stage of selection, a fixed number of 28 households per cluster selected with an equal probability systematic sampling from the newly created household listing. The detailed sampling procedure was available in the Ethiopian Demographic and Health Survey reports from measure DHS website (www.dhsprogram.com).

### Outcome variable

#### Consumption of iron rich foods

if a child aged 6–23 months consumed at least one iron rich foods item among the four food items (Egg, Meat, Organ meat, and Fish) at any time in the last 24 h preceding the interview declared as good consumption of iron rich foods; whereas, no consumption of iron rich foods in the last 24 h period preceding the interview declared as poor consumption of iron rich foods [11].

### Independent variables

From the standard 2016 EDHS dataset, age of the mothers’, educational status of mother and father, occupational status of the mother and father, mother’s religion, family size, wealth status, media exposure, child sex, and age of the child considered as the individual level independent variables. Whereas, residence and administrative region of the country were taken as community (cluster) level independent variables.

### Data management and analysis

The data were cleaned using STATA version 16.0/MP software and Microsoft Excel. Sample weighting was performed for further analysis.

### Spatial autocorrelation and hot spot analysis

Spatial autocorrelation (Global Moran’s I) statistic measure used to assess spatial heterogeneity for consumption iron rich foods among children age 6–23 months. Moran’s I values close to − 1 indicates poor consumption of iron rich foods which is dispersed, close to + 1 indicates clustered, and if Moran’s I value zero indicates randomly distributed [12]. A statistically significant Moran’s I value (*p* <  0.05) had a chance to reject the null hypothesis which indicates the presence of spatial autocorrelation. Hot spot analysis (Getis-Ord Gi* statistic) z-scores and significant *p*-values gave the features with either hot spot or cold spot values for the clusters spatially.

### Spatial interpolation

The spatial interpolation technique used to predict poor consumption of iron rich foods among children aged 6–23 months for un-sampled areas in the country. For the prediction of un sampled Enumeration Areas, we used geo-statistical Empirical Bayesian Kriging spatial interpolation techniques using ArcGIS 10.7(ESRI Inc., Redlands, CA, USA, version 10.7) software. Empirical Bayesian Kriging relaxes the assumption of the Gaussian distribution of the observed semi-variogram in the input data which rarely holds in practice. Empirical Bayesian Kriging interpolation works by generating a new simulated semi-variogram at each location from the estimated semi-variogram from the input data. The weight of the new simulated semi-variogram was calculated by Bayes’ rule (Krivoruchko, 2012 #1) [13].

### Spatial scan statistics

We employed Bernoulli based model spatial scan statistics to determine the geographical locations of statistically significant clusters for poor consumption of iron rich foods among children aged 6–23 months using Kuldorff’s SaTScan version 9.6 software [14]. The scanning window that moves across the study area in which children aged 6–23 months with poor consumption of iron rich foods taken as cases and those good consumption taken as controls to fit the Bernoulli model. The default maximum spatial cluster size of less than 50% of the population used as an upper limit, allowing both small and large clusters to be detected, and ignored clusters that contained more than the maximum limit with the circular shape of the window. Most likely clusters identified using *P*-values and log-likelihood ratio tests based on the 999 Monte Carlo replications.

### Multilevel mixed-effects logistic regression analysis

The nature of EDHS dataset was Hararichial which is children nested in household and household is nested in clusters. Therefore, the observations within the cluster correlated (dependant) that violates the assumption of independence. In correlated data, fitting models by ignoring these correlations will bias the parameter estimates. The correlation within the cluster diagnosed by intraclass correlation (ICC) value. If the ICC value greater than 0.25 [15] was reasonable to fit multilevel mixed-effects logistic regression model. The intraclass correlation (ICC) calculated by the variation between and within-cluster variation (ICC = ϭ^2^/ (ϭ^2^ + π^2^/3). We fitted four models, the null model without independent variables, model I with only individual-level variables, model II with only community-level variables, and model III both the individual level and community level variables. The model was fitted by a STATA version 16/MP*“xtmelogit”* command for multilevel mixed-effects logistic regression analysis. We used the Log-Likelihood Ratio (LLR) for model comparison. The highest log-likelihood was taken as the best fit model. Median Odds Ratio (MOR = exp. [squrt (2ψΦ^− 1^ (3/4))], where ψΦ^− 1^ = variance of the cluster) was employed to observe the heterogeneity between clusters. The MOR quantifies the variation between clusters (the second-level variation) by comparing two persons from two randomly chosen different clusters. Considering the two persons with the same covariates, chosen randomly from two different clusters; If we select on child randomly from a cluster the median value of the odds ratio at the high-risk cluster compared to low risk cluster for the consumption of iron rich foods. The measure is always greater than or equal to 1. If the MOR is 1, there is no variation between clusters (no second-level variation). If there is considerable between-cluster variation, the MOR will be large. The measure is directly comparable with fixed-effects Odds Ratio [16]. Percentage Change Variation (PCV) measures the total variation attributed by individual and community level factors in the multilevel model as compared to the null model which is calculated by the difference from variance in the null model to variance in the full model divided by variance in the null model.

### Ethical consideration

The authors, submitted concept note to DHS Program/ICF International Inc., letter of permission was waived from the International Review Board of Demographic and Health Surveys (DHS) program data archivists to download the dataset for this study.

## Results

### Characteristics of the study participants

From the EDHS dataset, a total of 3055 children aged 6–23 months were included in this study. More than half (53%) of the children were females. Eighteen and 36.6% of the children were between the age of 6–8 and 12–17 months. The mean ± SD of the age of the children was 14 ± 5 months. The majority (67.50%) of the children were born from mother’s age between 20 and 34 years. The mean ± SD age of the mothers was 28 ± 6 years. Approximately 61% of the mothers and 45% of fathers had no formal education. Around 44% of the children born from poor household wealth status (Table 1).

**Table 1:** Sociodemographic characteristics of the respondents and study children aged 6*–*23 months EDHS 2016, Ethiopia. (*n* = 3055)

Variables		Frequency (n)	Weighted Percentage (%)
Mother’s age in years	< 20	369	12.08
	20–34	2062	67.50
	35–49	624	20.42
Marital status	Married	2898	94.84
	Not married	157	5.16
Religion	Orthodox	1049	34.35
	Muslim	667	21.83
	Protestant	1236	40.45
	Others	103	3.37
Mother’s education	No education	1865	61.03
	Primary education	935	30.62
	Secondary education and above	255	8.53
Husband’s education	No education	1291	44.55
	Primary education	1198	41.34
	Secondary education and above	408	16.10
Mother occupation	Working	1299	42.52
	Not working	1756	57.48
Father occupation	Working	2833	92.74
	Not working	222	7.26
Family size	< Five	2043	66.87
	≥ Five	1012	33.13
Child age in months	6–11	1066	34.88
	12–17	1118	36.59
	18–23	871	28.53
Child sex	Male	1433	46.90
	Female	1622	53.10
Media exposure	No media exposure	2043	68.25
	Had media exposure	950	31.75
Household wealth	Poor	1350	44.18
	Middle	683	22.37
	Rich	1022	33.44
Residence	Rural	2684	87.85
	Urban	371	12.15
Region	Tigray	227	7.45
	Afar	30	0.97
	Amhara	560	18.34
	Oromia	1341	43.91
	Somali	123	4.03
	Benishangul	32	1.04
	SNNPR	632	20.68
	Gambela	7	0.24
	Harari	8	0.25
	Addis Adaba	81	2.65
	Dire Dawa	14	0.44
**Total**		**3055**	**100**

### Consumption of iron rich foods among children aged 6–23 months in Ethiopia

Overall, 21.41% (95% CI: 19.9–22.9) of children aged 6–23 months had good consumption of iron rich foods in Ethiopia. Consumption of egg was the most taken food items among children aged 6–23 months. Consumption of fish or shellfish foods were the least taken food (Table 2).

**Table 2:** Consumption of iron rich foods among children aged 6*–*23 months, 2016 EDHS, Ethiopia

S. No	Food groups interviewed in the last 24 h.	Consumption status	
		Good (%)	Poor (%)
1	Has the child consumed eggs in the last 24 h?	16.81	83.19
2	Has the child consume meat (beef, pork, lamb, chicken, etc.) in the last 24 h?	5.95	94.05
3	Has the child consumed organ meat (liver, …) in the last 24 h?	3.89	96.11
4	Has the child consume fish or shellfish in the last 24 h?	1.31	98.69
**Overall consumption of iron rich foods among children age 6–23 months**		**21.41**	**78.59**

### Spatial distribution of poor consumption of iron rich foods among children aged 6–23 months in Ethiopia

**Fig. 1:**
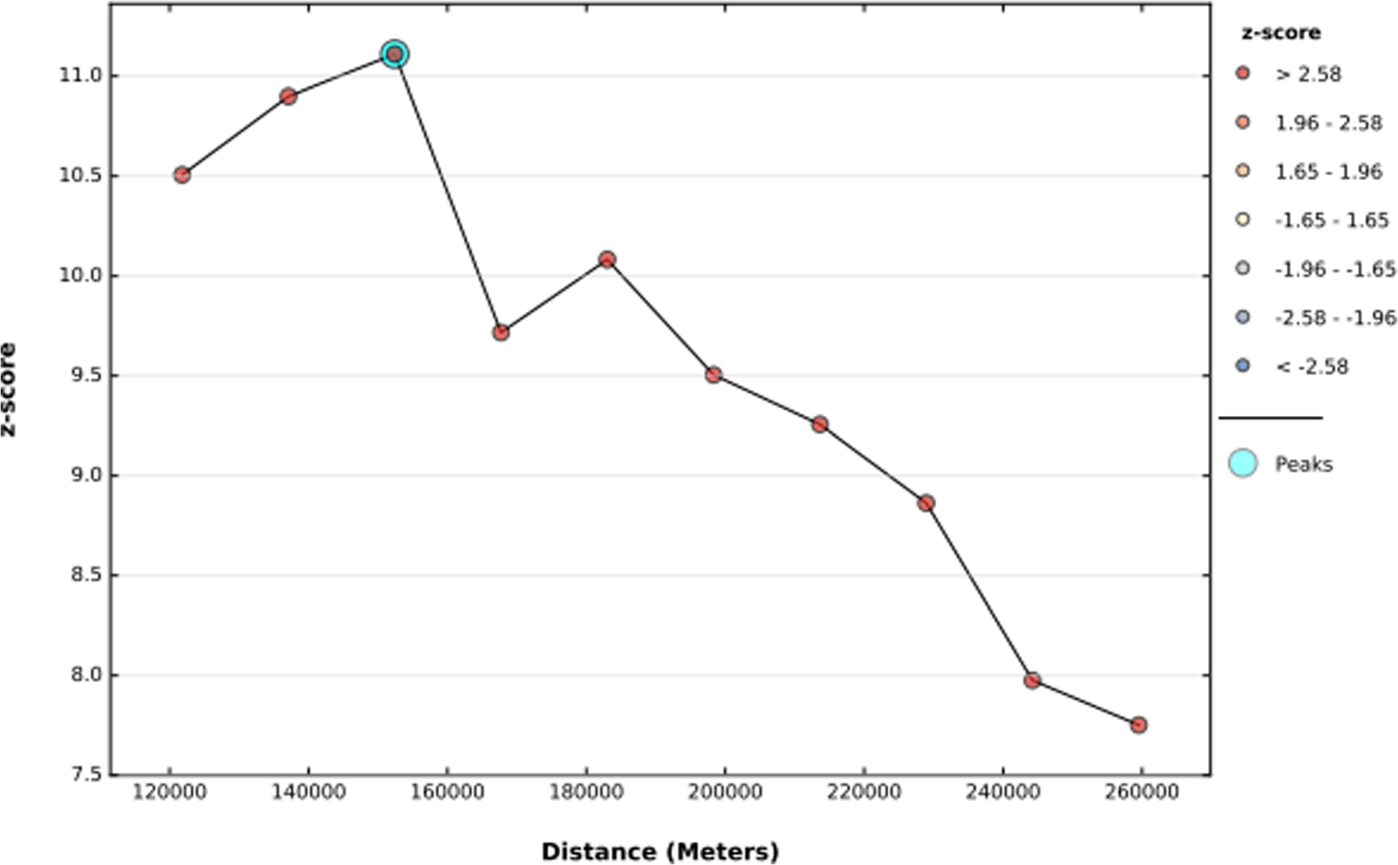
Incremental spatial autocorrelation of poor consumption iron-rich foods among children age 6–23 months in Ethiopia, 2016, EDHS

**Fig. 2:**
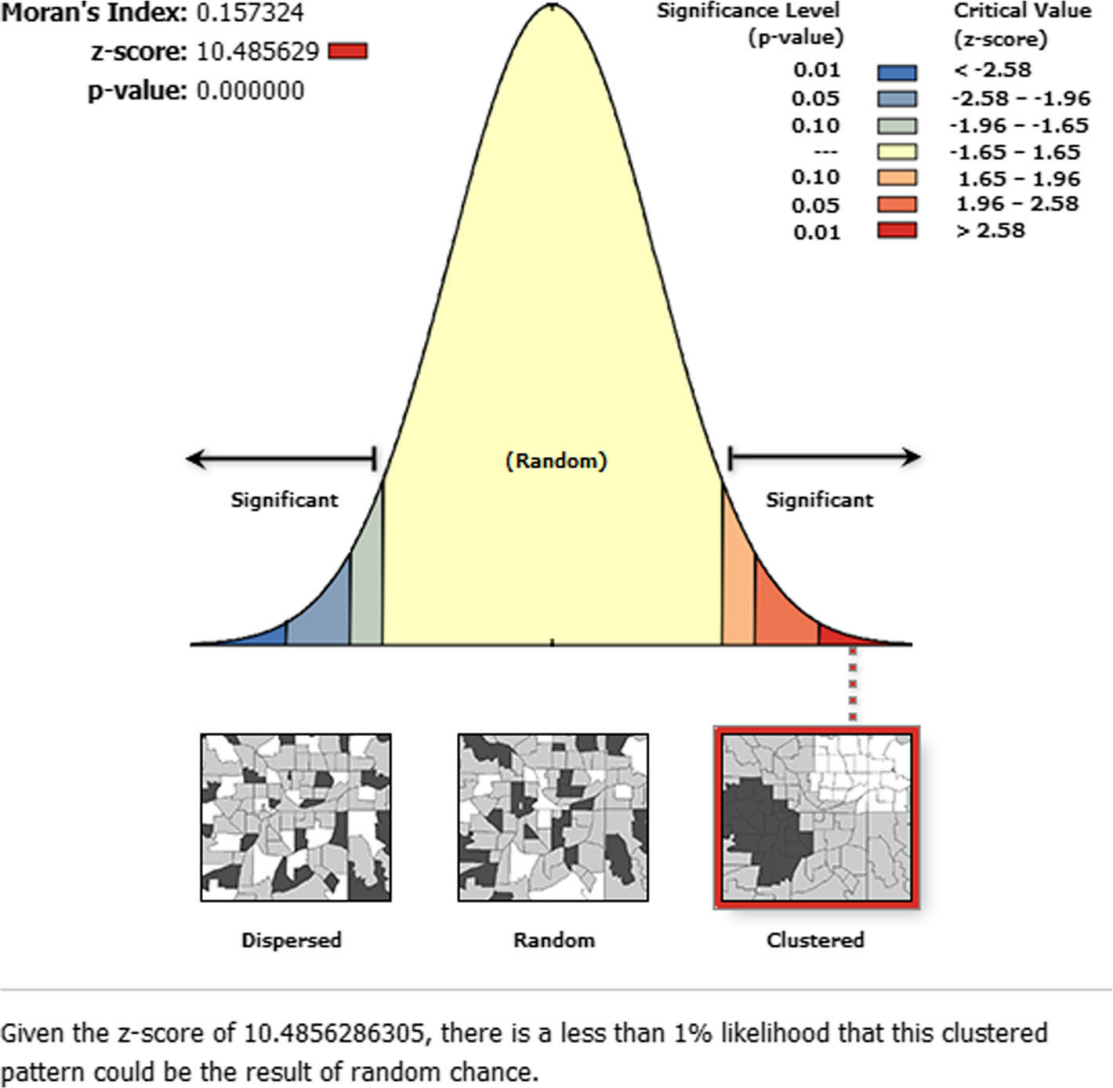
Spatial autocorrelation report of poor consumption iron-rich foods among children age 6–23 months in Ethiopia, 2016, EDHS

The incremental spatial autocorrelation evidenced that with 10 distance bands starting from 121.8 km (km) iron rich foods consumption clustering was detected at 152.4 km distance. Statistically, significant z-score indicates at 152.4 Km distance spatial clustering of iron rich foods consumption was most pronounced (Fig. 1). The spatial distribution of poor consumption of iron rich foods among children aged 6–23 months were non-random in Ethiopia (Global Moran’s I = 0.16, z-score = 10.45, *P*-value < 0.001). This evidenced that spatial hot spot and cold spot clustering identified in the regions of Ethiopia. Given the z-score of 10.48, there was less than 1% likelihood that this high-clustered pattern could be the result of random chance (Fig. 2).

### Hot spot (Getis-Ord Gi*) analysis

**Fig. 3:**
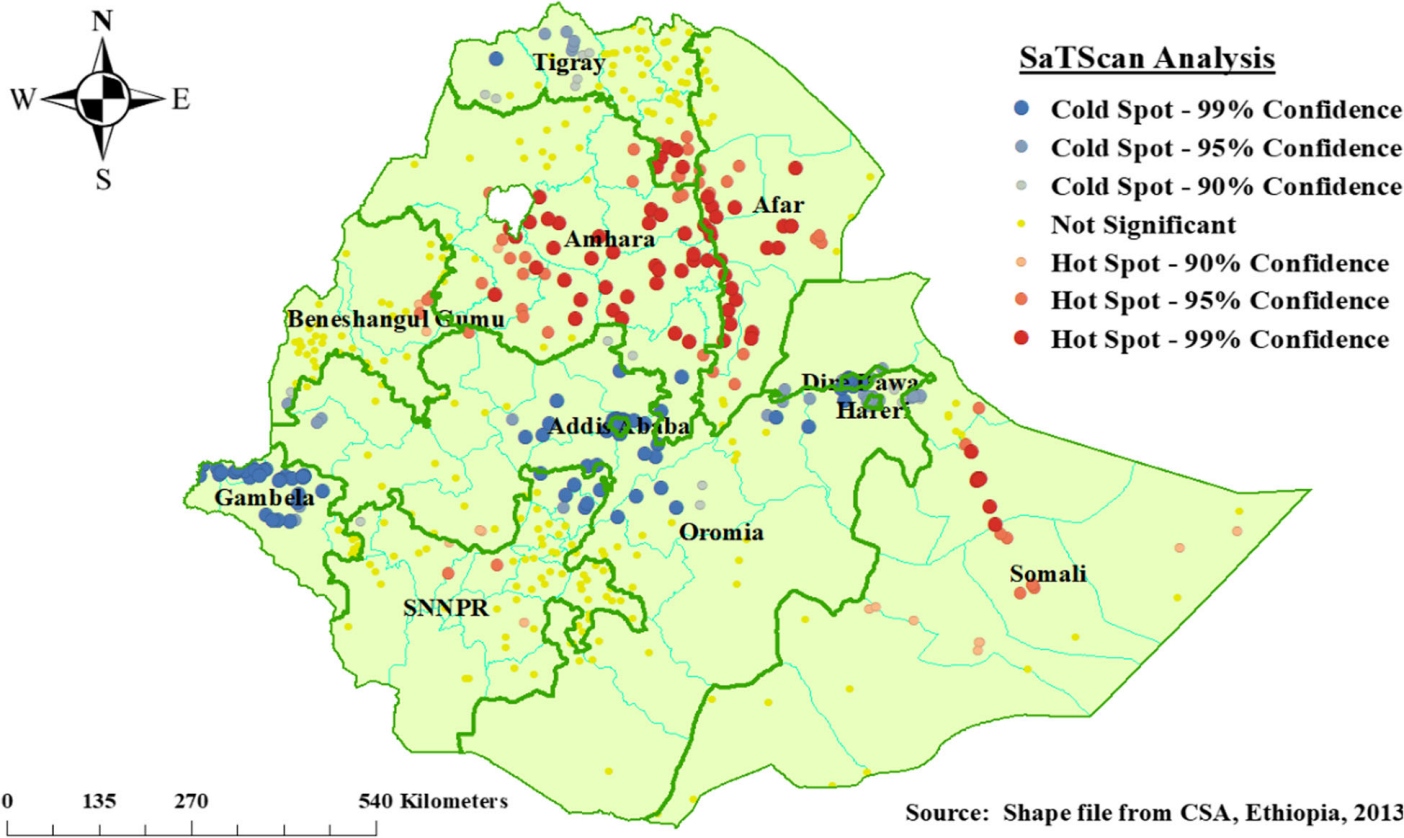
Hot spot analysis of poor consumption iron-rich foods among children age 6–23 months, Ethiopia, 2016, EDHS

The spatial hot spot analysis was predicted using incremental spatial autocorrelation maximum pick distance value 152.4 km. As shown in Fig. 3 below, the red color is intense clustering of high (hot spot) proportion with poor consumption of iron rich foods among children aged 6–23 months in Ethiopia. The prevalence of poor consumption of iron rich foods among children aged 6–23 months were clustered at Southeastern Amhara, Southern part of Afar, and Northern part of Somali Regional States of Ethiopia; whereas, Addis Ababa, Gamebela, Central Oromia, and Northern part of Southern Nations, Nationalities, and People’s Regional states of Ethiopia were less risk area for poor consumption of iron rich foods among children aged 6–23 months (Fig. 3).

### Spatial SaTScan analysis

**Fig. 4:**
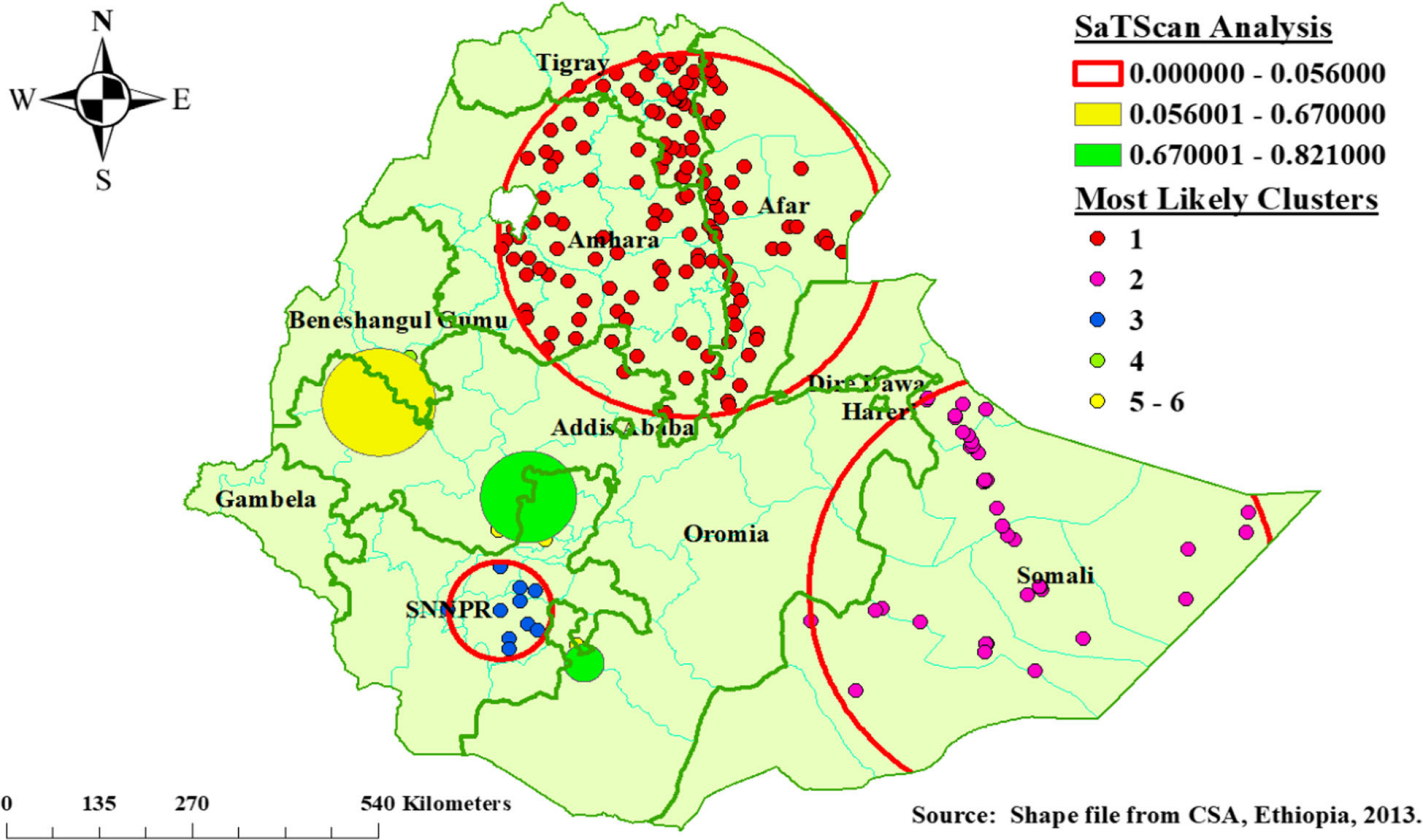
SaTScan analysis for poor consumption of iron-rich foods among children age 6–23 months, EDHS, 2016, Ethiopia

As shown in Fig. 4 below, the red window indicates significant clusters. A total of 190 significant clusters identified. Among the significant clusters, 142 were most likely and the other 48 were secondary and tertiary clusters. The most likely (primary) clusters were located at 11.626646 N, 39.666950 E within 278.08 km radius in Afar, the Southeastern part of Tigray, and the Eastern part of Amhara Regional States of Ethiopia. Children aged 6–23 months living in the most likely cluster were 21% more likely vulnerable to poor consumption of iron rich foods than outside the window (RR = 1.21, LLR = 40.107, *P*-value < 0.001) (Table 3 and Fig. 4).

**Table 3:** Significant SaTScan spatial scan clusters for poor consumption of iron rich foods among children aged 6–23 months, 2016 EDHS, Ethiopia

Cluster type	Significant Enumeration Areas (clusters) detected	Coordinates /Radius	Populations	Cases	RR	LLR	***P***-value
**Primary**	496, 189, 611, 571, 191, 345, 478, 254, 389, 591, 18, 200, 241, 455, 401, 354, 332, 368, 616, 344, 249, 348, 55, 617, 97, 351, 488, 544, 442, 547, 545, 300, 66, 136, 570, 627, 276, 449, 460, 128, 599, 620, 143, 334, 176, 38, 392, 205, 79, 542, 310, 283, 499, 178, 10, 267, 199, 637, 511, 102, 130, 160, 37, 206, 132, 172, 295, 135, 120, 421, 424, 427, 440, 456, 538, 632, 510, 596, 384, 572, 512, 336, 628, 605, 482, 550, 237, 94, 24, 484, 220, 201, 163, 575, 75, 403, 430, 152, 327, 158, 585, 350, 423, 99, 298, 623, 167, 579, 235, 425, 564, 429, 312, 80, 4, 127, 73, 531, 196, 355, 362, 169, 129, 382, 322, 551, 230, 640, 226, 375, 431, 51, 604, 474, 263, 156, 341, 218, 516, 121, 481, 188	11.626646 N, 39.666950 E/ 278.08 km	663	597	1.21	40.12	< 0.001
**Secondary**	92, 490, 543, 492, 171, 198, 146, 95, 85, 358, 138, 164, 497, 278, 521, 588, 458, 553, 77, 629, 214, 318, 251, 573, 187, 239, 116, 22, 33, 568, 277, 527, 269, 556, 630, 64, 439, 480	6.745 N, 44.259 E / 360.64 km	201	191	1.23	23.54	< 0.001
**Tertiary**	406, 434, 450, 505, 141, 180, 86, 503, 306, 470	6.448207 N, 37.165249 E / 74.90 km	51	50	1.26	8.95	< 0.05

### Interpolation of poor consumption iron rich foods among children age 6–23 months

**Fig. 5:**
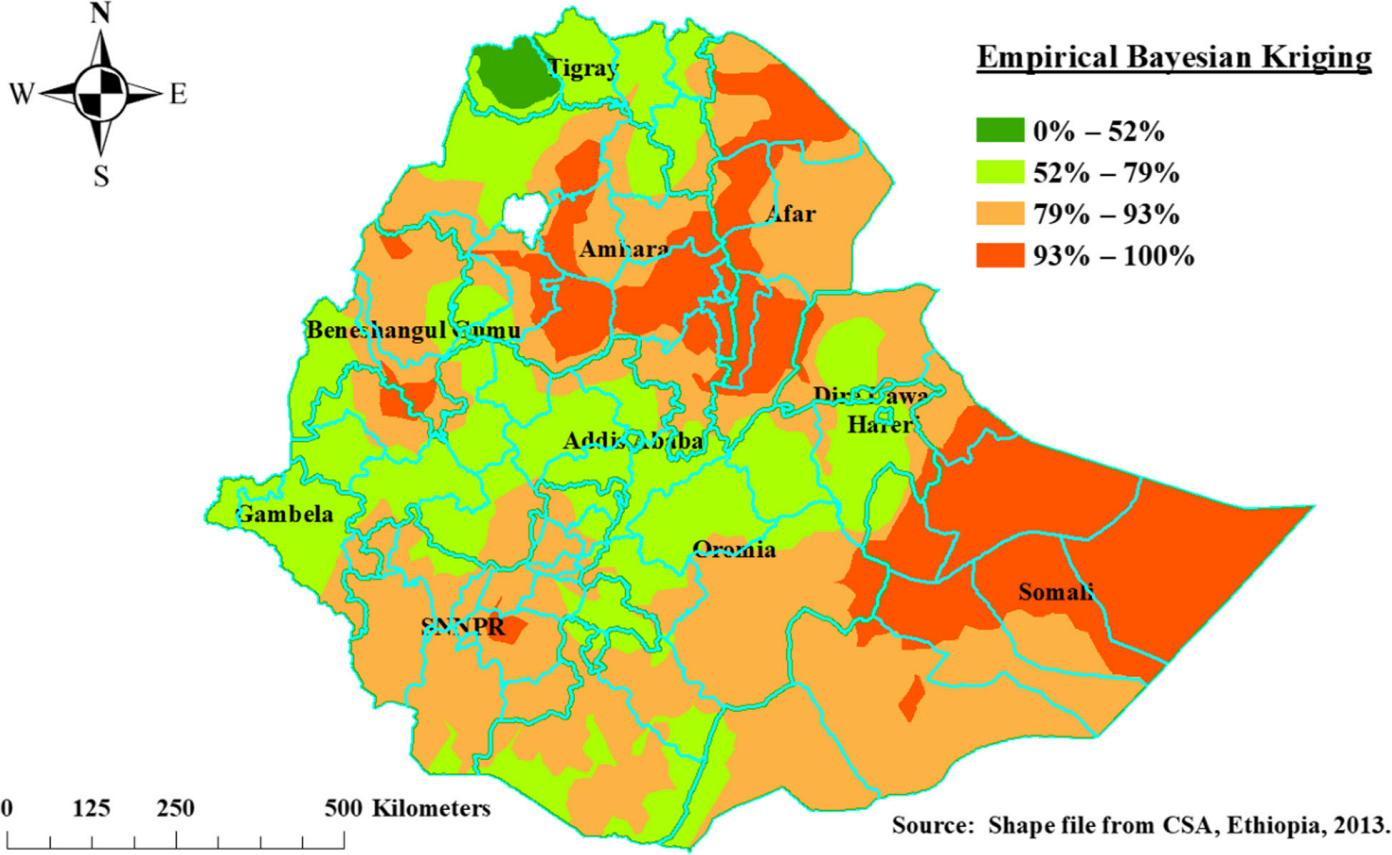
Empirical Bayesian Kriging interpolation of poor consumption iron-rich foods among children age 6–23 months, EDHS, 2016, Ethiopia

We employed an Empirical Bayesian Kriging interpolation method. The interpolation result revealed that children aged 6–23 months were venerable to poor consumption of iron rich foods in most part of Ethiopia. Relatively good consumption of iron rich foods among children aged 6–23 months were observed at Tigray, Gambela, Addis Ababa, and Central Oromia Regional States of Ethiopia (Fig. 5).

### Multivariable multilevel mixed-effects logistic regression analysis

In the multilevel mixed effects logistic regression model, the full model contains both individual and community-level factors that were the best fit model (LLR = − 1223.36). From the null model, 27% of the variability of the odds of iron rich foods consumption accounted for intra class correlation (ICC =0.27). As a rule of thumb, the intra class correlation (ICC) variation greater than 0.25 indicates that intra-cluster correlation. The dependency within the cluster accounted for multilevel mixed effects model which is reliable by adjusting individual and community level factors. The experience of iron rich foods consumption was heterogeneous between clusters which were explained by Median Odds Ratio (MOR). In the full model, the MOR was 2.38 which means if we select one child in different cluster live in less risk cluster had 2.38 times more likely to consume iron rich foods than children live in high risk cluster (MOR = 2.38,95% CI: 1.91–4.34) (Table 2).

### Individual and community level factors for good consumption of iron rich foods

In multivariable multilevel mixed-effects logistic regression analysis, child age, educational status of the mother, wealth index, and region were statistically significant factors for good consumption of iron rich foods in Ethiopia.

After adjusting other variables, children aged between 12 and 17 months was 90% more likely to consume iron rich foods than children aged between 6 and 11 months (AOR = 1.90, 95% CI: 1.45–2.49). Besides, the odds of good consumption of iron rich foods among children aged between 18 and 23 months were 2.05 times higher than children aged between 6 and 11 months (AOR = 2.05, 95% CI: 1.55–2.73).

The odds of having good consumption of iron rich foods were 2.26 times higher than children who born from mother with secondary and above education level as compared to children born from non-educated mother’s (AOR = 2.26, 95% CI: 1.47–3.46). Similarly, children who born from mother with primary education was 42% times more likely to have good consumption of iron rich foods than children born from non-educated mother (AOR = 1.42, 95% CI:1.06–1.87).

Children aged 6*–*23 months born from middle household wealth status were 83% more likely to consume iron rich foods as compared to children born from poor household wealth status (AOR = 1.83, 95% CI: 1.31–2.57). In addition, children born from rich household wealth status were 49% more likely to have good consumption of iron rich foods as compared to the reference category (AOR = 1.49, 95% CI: 1.04–2.13).

Children aged 6–23 months from Afar and Amhara regional state of Ethiopia were 86% (AOR = 0.24, 95% CI: 0.09, 0.60) and 62% (AOR = 0.38, 95% CI: 0.17, 0.84) less likely to have good consumption of iron rich foods as compared to children live in Addis Ababa, Ethiopia respectively. Whereas, children live in Harari regional state of Ethiopia were two times more likely to consume iron rich foods than children living in Addis Ababa, Ethiopia (AOR = 2.11, 95CI: 1.02*–*4.39) (Table 4).

**Table 4:** Multivariable multilevel mixed effects logistic regression analysis factors associated with consumption of iron rich foods among children aged 6*–*23 months, 2016 EDHS, Ethiopia

Variables	Models			
	Null Model ^**a**^	Model II ^**b**^	Model III ^**c**^	Model IV ^**d**^
		AOR (95% CI)	AOR (95% CI)	AOR (95% CI)
**Mother’s age**				
< 20 Years		1		1
20–34 years		1.07 (0.72–1.58)		1.03 (0.69–1.53)
35–49 Years		1.01 (0.60–1.70)		0.97 (0.57–1.63)
**Child age**				
6–11 months		1		1
12–17 months		1.92 (1.46–2.52) ***		1.90 (1.45–2.49) ***
18–23 months		2.14 (1.61–2.85) ***		2.05 (1.55–2.73) ***
**Religion**				
Orthodox		1		1
Muslim		0.97 (0.67–1.42)		0.89 (0.57–1.39)
Protestant		0.85 (0.62–1.17).)		0.94 (0.63–1.40)
Others ¥		1.21 (0.58–2.51)		1.06 (0.50–2.24)
**Mother education**				
No education		1		1
Primary education		1.59 (1.19–2.12) ***		1.42 (1.06–1.87) *
Secondary and above		2.70 (1.77–4.12) ***		2.26 (1.47–3.46) ***
**Father education**				
No education				
Primary education		1.15 (0.87–1.53)		0.99 (0.74–1.32)
Secondary and above		1.36 (0.93–1.97)		1.13 (0.77–1.64)
**Mother’s occupation**				
Not working		1		1
Working		1.21 (0.95–1.53)		1.18 (0.93–1.50)
**Father occupation**				
Not working		1		1
Working		1.20 (0.79–1.81)		1.23 (0.81, −1.86)
**Media exposure**				
No media exposure		1		1
Has media exposure		1.42 (1.08–1.88) *		1.30 (0.98–1.72)
**Birth order**				
1		1		1
2–4		0.73 (0.53–1.01)		0.75 (0.54–1.03)
> = 5		0.94 (0.53–1.67)		0.96 (0.54–1.69)
**Family size**				
< 5		1		1
≥ 5^+^		0.85 (0.50–1.43)		0.82 (0.48–1.37)
**Wealth index**				
Poor		1		1
Middle		1.94 (1.38, − 2.71) ***		1.83 (1.31–2.57) ***
Richer		1.73 (1.23–2.40) ***		1.49 (1.04–2.13) *
**Residence**				
Urban			1	1
Rural			0.39 (0.28–0.54)	0.71 (0.46–1.10)
**Region**				
Addis Adaba 0.79 (0.44, 1.40) 0.66 (0.33, 1.32)			1	1
Tigray			1.27 (0.71–2.27)	1.50 (0.76–2.98)
Afar			0.15 (0.06–0.32) ***	0.24 (0.09–0.60) **
Amhara			0.30 (0.15–0.60) ***	0.38 (0.17–0.84) *
Oromia			1.25 (0.69–2.26)	1.47 (0.73–2.94)
Somali			0.25 (0.14–0.51)	0.49 (0.22–1.07)
Benishangul			0.89 (0.46–1.70)	1.09 (0.51–2.33)
SNNPR			0.68 (0.37–1.25)	0.83 (0.41–1.71)
Gambela			1.41 (0.76–2.60)	1.72 (0.81–3.66)
Harari			1.76 (0.95–3.25)	2.11 (1.02–4.39) *
Dire Dawa			0.71 (0.38–1.24)	0.90 (0.42–1.97)
**Random effects**				
**ICC%**	**0.27**			
**MOR (95%CI) 2.88 (1.91–4.34)**		**2.70 (1.79–4.08)**	**2.08 (1.59–2.73)**	**2.38 (1.66–3.41)**
**PCV%**	**0**	**12%**	**52%**	**33%**
**Model compression**				
**Log likelihood ratio**	**− 1458.724**	**− 1256.25**	**− 1372.049**	**−1223.363**

NB: * = Significant at *P*-value 0.05, ** = Significant at *P*-value 0.01, *** = Significant at *P*-value 0.001, *CI* Confidence Interval, *AOR* Adjusted Odds Ratio, *ICC* Intraclass Correlation, *MOR* Median Odds Ratio, *PCV* Percentage Change Variation, ¥ = Catholic, Traditional, and other

^a^ = Adjusted for individual and community level variable, ^b^ = Adjusted for community-level variable, ^c^ = Adjusted for individual-level variable, ^d^ = Full model

## Discussion

Low intake of bioavailable iron and iron rich foods is the major cause of the high prevalence of iron-deficiency anemia among children aged 6–23 months in developing countries [17].

This study revealed that only 21.41% (95%CI: 19.9*–*22.9) of children aged 6–23 months had good consumption of iron rich foods. Egg consumption was the highest consumed food among the study participants. This study is similar to the 2015 Demographic and Health Survey report of Rwanda which was 20% [18] and higher than the 2011 Ethiopian Demographic and Health Survey report (13%) [19]. The result of this study was lower than DHS report of Kenya in 2014 (33%), Malawi in 2015 (38%), Uganda in 2016 (40%), Zimbabwe in 2016 (46%), and Zambia in 2013/2014 (49%) [20–24]. The report of Ethiopian Demographic Health Survey on iron rich foods consumption among children aged 6–23 months was low as compared to other East African countries. The possible explanation might be a socio-demographic, cultural, and behavioral difference across the countries for the child feeding practice. As well it might be the different cultural practice among children feeding practice across the country. Besides, in Ethiopia animal source foods are consumed during special societal occasions since they are considered as luxury food rather than an essential part of daily children’s diet; This qualitative study revealed that the consumption of animal source foods among children aged 6–23 months was very low and the home-reared livestock and their products were mainly used for market purposes [25].

The spatial distribution of iron rich foods consumption among children aged 6–23 months was non-random in Ethiopia. Children aged 6–23 months lived in Southeastern Amhara and Tigray, Southern part of Afar, and Northern part of Somali regional state of Ethiopia was high risk for poor consumption of iron rich foods. As well, in line with spatial hot spot area, the spatial scan statistics revealed that children aged 6–23 months lived in the most likely cluster were 21% more likely vulnerable to poor consumption of iron rich foods than outside the window. The possible spatial heterogeneity across the regions of Ethiopia might be the dietary preference, practice to complementary feeding, socioeconomic status, and demographic factor such as pastoralist region (Afar and Somali). This spatial variation might be also availability, accessibility, and affordability of iron rich foods across regions of Ethiopia. Also, the difference in spatial heterogeneity of iron rich foods consumption might be lack of nutrition knowledge, high cost of animal source foods, low household income, low milk production, the poor linkage between health and agriculture sectors, and social norms and beliefs across the regions of Ethiopia [25].

From the multilevel mixed-effects logistic regression analysis, child age, educational status of the mother, wealth index, and regions of Ethiopia were statistically associated with good consumption of iron rich foods. The likelihood of iron rich foods consumption among children aged between 12 and 17 and 18–23 months were 90% and two times higher than children aged 6–11 months respectively. Even though we could not get studies on iron rich food consumption among children aged 6–23 months, it is fair to discuss with studies conducted on anemia. From a study conducted in Northeast Ethiopia, children aged 6–11 months were 4.5 times more likely to be anemic as compared to older children [26]. From previous studies prevalence of iron deficiency anemia was high among children aged 6–11 months [4, 27–29]. Therefore, this study supports the fact that iron deficiency anemia was high among those age group children. The possible justification might be children in the early period of six months would not start complementary foods due to behavioral and cultural practice among the study participants.

Children born from educated mother had a high intake of iron rich foods as compared to children born from non-educated mothers. The odds of iron rich foods consumption among children aged 6–23 months born from mothers with primary education were 42% more likely to consume iron rich foods than children born from non-educated mothers. As well, the likelihood of iron rich foods consumption among children born from mothers with secondary education and above was two times higher than children born from non-educated mothers. The prevalence of anemia among children aged 6–23 months born from educated mothers were low. The previous study in Ethiopia showed that children born from non-educated mothers were 2.6 times more likely to be anemic than children born from educated mothers [27]. Another study also evidenced that children born from non-educated mothers were four times anemic [26]. The possible explanation might be educated mothers might know the importance of complementary feeding practice, dietary consumption of iron-rich foods for their child as well they might have knowledge on the cause of iron deficiency anemia [30]. Besides educated mothers might have a positive influence on the cultural practice of child feeding practice. Educated mothers follow scientifically proved feeding practice and a strong predictor for good nutritional feeding outcomes for the children.

The wealth index status of the household affects iron rich foods consumption status. Children from middle household wealth status were 83% more likely to have good consumption of iron rich foods as compared to poor household wealth index status. This might be due to high household wealth status could afford and access iron rich foods for their child. Food insecure and poor wealth status of the households were associated with lack of micronutrient-rich food consumption which may lead to iron deficiency anemia [31–33].

Moreover, the regions of Ethiopia were statistically significant with iron rich foods consumption. Children aged 6–23 months living in Afar and Amhara regional states of Ethiopia were less likely to consume iron rich foods as compared to children living in Addis Ababa, whereas children live in Harari were more likely to consume iron rich foods. The possible explanation might be in line with spatial heterogeneity as well as socio-cultural practice on child feeding practice across the regions of Ethiopia.

### Strengths and limitations of this study

First, this study was conducted by using a nationally representative demographic and health survey dataset which may give better generalization. Secondly, accounting spatial heterogeneity was a strength for this study. Thirdly, accounting clustering effect using a mixed-effects model was also another strength for this study.

As well this study had some limitations. The first limitation was 2016 Ethiopian Demographic Health Survey dataset lacks some spatial coordinates in the Somali region which may mislead to spatial statistics estimate. Since the data were collected using face to face interview it might prone to recall and social desirability bias. The drawback of the secondary nature of data was inevitable.

### Conclusion and recommendation

A significant number of children aged 6–23 months hadn’t good consumption of iron rich foods. The spatial distribution of iron rich foods in Ethiopia was non-random. Spatial clustering of low consumption of iron rich foods was identified at Southeastern Amhara region, Eastern part of Tigray, the Southern part of Afar region, and some part of Northern Somali regional states of Ethiopia. Child age, educational status of the mother, wealth index and regions of Ethiopia were significantly associated with iron rich foods consumption among children age 6–23 months. Responsible stakeholders should give primary attention to children living in the hotspot areas of Ethiopia. Besides, complementary iron rich foods consumption should be promoted particularly in the age group of 6–11 months of children.

## Data Availability

The data was available from the corresponding author and we can provide a reasonable request.
